# Transdiagnostic Investigation of Impulsivity in Alcohol Use Disorder and Binge Eating Disorder With Eye-Tracking Methodology—A Pilot Study

**DOI:** 10.3389/fpsyt.2019.00724

**Published:** 2019-10-18

**Authors:** Kathrin Schag, Magdalena Rauch-Schmidt, Friederike Wernz, Stephan Zipfel, Anil Batra, Katrin E. Giel

**Affiliations:** ^1^Department of Psychosomatic Medicine and Psychotherapy, University Hospital Tübingen, Tübingen, Germany; ^2^Competence Center for Eating Disorders Tübingen (KOMET), Tübingen, Germany; ^3^Department of Psychiatry and Psychotherapy, Section Addiction Medicine and Addiction Research, University Hospital Tübingen, Tübingen, Germany

**Keywords:** alcohol, addiction, binge eating, eye tracking, food, impulsivity

## Abstract

**Objective:** Patients with alcohol use disorder (AUD) and patients with binge eating disorder (BED) are characterized by increased impulsivity, i.e. increased reward sensitivity and diminished response inhibition. In this pilot study, we compare both disorders directly concerning impulsivity using disorder-specific stimuli to gain insight into the relationship of both disorders and underlying mechanisms.

**Methods:** We compared eye movements of 23 women with BED (age *M* = 40.9), 21 participants with AUD (13 females, 8 males, age *M* = 46.6), and age- and sex-matched control groups (BED-CG and AUD-CG, respectively). We measured reward sensitivity with the free exploration paradigm and response inhibition with the modified antisaccade paradigm. We presented disorder-specific stimuli vs. neutral stimuli, i.e. food stimuli in the BED and BED-CG and alcohol stimuli in the AUD and AUD-CG.

**Results:** BED and BED-CG initially fixated more often on food stimuli vs. neutral stimuli, whereas AUD and AUD-CG initially fixated more often on neutral stimuli vs. alcohol stimuli. AUD showed shorter dwell times on both stimulus categories in comparison with the other groups. When saccades towards stimuli should be inhibited, BED made more errors in first saccades for both stimulus categories in comparison with AUD-CG and in second saccades particularly for food stimuli in comparison with all other groups, whereas AUD did not differ from the control groups.

**Conclusions:** This pilot study indicates that food and alcohol stimuli are at the first sight differently processed. Moreover, patients with BED and with AUD seem to process disorder-specific stimuli differently. Whereas patients with AUD avoid stimuli generally, patients with BED predominantly show deficits in inhibitory control.

## Introduction

Patients with binge eating disorder (BED) and patients with severe substance use disorders (SSUD) show similarities in the core pathology of their disorders and underlying neuropsychological functions (1–3). Patients with SSUD and with BED report increased craving concerning the respective desired substance and a subjective loss of control during substance consumption [e.g., (4)]. While SSUD have for a long time been recognized as addiction, BED is a comparably novel diagnosis and has recently been controversially discussed to be conceptualized as a “food addiction,” as it overlaps particularly with behavioral addictions (5, 6).

Both, patients with SSUD and BED show impairments in impulsivity, a personality trait featured by increased reward sensitivity and decreased inhibitory control (7–9). According to several reviews, behavioral and self-report studies, trait impulsivity is increased in BED and SSUD (10–14) and predicts onset or symptom severity (15–17). Beyond increased trait impulsivity, patients affected by SSUD or BED seem to show even stronger impairments when processing disorder-specific stimuli, i.e. food and alcohol like indicated by fMRI studies (18, 19) and systematic reviews (20, 21).

A direct comparison of both disorders is useful to clarify the role of impulsivity as a potential underlying mechanism in BED and SSUD, as well as the relationship between both disorders. Nevertheless, there are only few studies comparing both disorders directly, and they used disorder-unspecific stimuli (e.g. geometrical signs) and different behavioral paradigms to assess several aspects of impulsivity (22–24). Voon et al. (23) found that impulsivity in the sense of premature responding measured by a serial reaction time task is increased in patients with several addictive disorders in contrast to obese patients with and without BED. Mole et al. (22) found greater delay discounting in patients with alcohol addiction and obese patients with and without BED but impaired motor response inhibition measured by the Stop Signal task only in patients with alcohol addiction and obese patients without BED, but not in patients with BED. Voon et al. (24) showed that patients with BED have greater risk-taking concerning monetary rewards, but not to losses, similarly to patients with addictive disorders. Taken together, the results are mixed, but patients with addictions seem slightly more impaired.

In the current pilot project, we investigate participants with BED, participants with severe alcohol use disorder (AUD), and sex- and age-matched healthy participants concerning impulsivity towards disorder-specific stimuli, i.e. food or alcohol pictures vs. neutral pictures. To our knowledge, this is the first eye-tracking study that is comparing both patient groups directly. We used disorder-specific stimuli to gain more insight into the processing of food vs. drug rewards and to examine if disorder-specific stimuli lead to increased impairments. We investigated the groups with eye-tracking methodology by using two established paradigms, one assessing reward sensitivity and the second assessing inhibitory control (see 25). We expected that BED and AUD will show increased reward sensitivity and decreased inhibitory control concerning disorder-specific stimuli in comparison with their respective control group. Concerning neutral stimuli, we expected that AUD will show more impairments as BED according to prior research with disorder-unspecific stimuli (22–24).

## Materials And Methods

### Participants

Participants were recruited by flyers, circulars, or both in the study involved departments from the University Hospital Tübingen, Germany. We used 23 women from the sample of the study from Schag et al. (25) who fulfilled the criteria for a diagnosis of BED according to the *Diagnostic and Statistical Manual of Mental Disorders* (DSM)-5 (26) and a body mass index (BMI) of 27–45 kg/m^2^. In the AUD group, 21 participants (8 men and 13 women) with a diagnosis of severe AUD according to DSM-5 (26) have been assessed, which equates to an alcohol addiction according to DSM-IV (27). We assessed only females in the BED group and both sexes in the AUD group due to the respective sex ratios in the general population (28, 29). Twenty-three sex- and age-matched healthy participants with normal weight constituted the control group for BED (BED-CG), and 21 sex- and age-matched healthy participants constituted the control group for AUD (AUD-CG).

Exclusion criteria in all groups were assessed in a short checklist before the start of the study and comprised impaired and non-corrected vision, severe cognitive impairment, neurological syndromes, psychosis, bipolar disorder, and intake of psychotropic drugs except antidepressants. Specific exclusion criteria were SSUD in the BED group, eating disorders in the AUD group, and mental disorders in general in the two control groups. Sample characteristics are presented in **Table 1**.

**Table 1:** Sample characteristics (M ± SD) in the BED and AUD groups and the respective control groups.

	BED (*n* = 23)	BED-CG (*n* = 23)	AUD (*n* = 21)	AUD-CG (*n* = 21)	*p*	*Post hoc* group differences
Age	40.9 ( ± 11.3)	40.5 ( ± 11.6)	46.6 ( ± 11.2)	47.7 ( ± 12.4)	.088	–
BMI	35.5 ( ± 5.9)	22.5 ( ± 1.7)	23.7 ( ± 2.3)	24.2 ( ± 2.5)	.000	BED > BED-CG, AUD, AUD-CG
BIS-11 score	67.6 ( ± 10.2)	60.3 ( ± 7.6)	58.9 ( ± 9.3)	54.8 ( ± 7.7)	.000	BED > BED-CG, AUD, AUD-CG
BDI II score	16.6 ( ± 11.8)	1.7 ( ± 2.2)	10.2 ( ± 8.1)	1.7 ( ± 2.3)	.000	BED, AUD > BED-CG, AUD-CG

This study was carried out in accordance with the recommendations of the ethics committee of the Medical Faculty at the Eberhard Karls University Tübingen, Germany with written informed consent from all subjects in accordance with the Declaration of Helsinki. The protocol was approved by the ethics committee of the Medical Faculty at the Eberhard Karls University Tübingen, Germany.

### Experimental Paradigms and Stimulus Material

Eye tracking with similar paradigms has been used in addiction (30, 31) and eating disorder research (32, 33) before. The same laboratory setup and eye-tracking devices have been used as already described in Schag et al. (25). We used the free exploration paradigm to assess reward sensitivity for disorder-specific stimuli (25, 34). In this paradigm, participants are instructed to visually explore 24 stimulus pairs, consisting of food vs. neutral stimuli in the BED and BED-CG groups and consisting of alcohol vs. neutral stimuli in the AUD and AUD-CG groups. Each stimulus pair was presented for 3,000 ms preceded by a fixation cross for 2,000 ms. As dependent variables, we analyzed the frequency of initial fixation positions on the disorder-specific vs. neutral stimuli, indicating early and unconscious attentional orientation and the dwell time (ms) on the disorder-specific vs. neutral stimuli that is addressing ongoing and deliberate attention. As some participants did often look on the gray background instead of the stimuli, this time was additionally considered.

To assess response inhibition, we used the modified antisaccade paradigm (25). There, participants are instructed to look away from the disorder-specific vs. neutral stimuli that are presented in random order as single pictures at the left or right side of the screen for 1,000 ms each, resulting in 96 trials. Before each stimulus presentation, a fixation cross (1,250 ms) followed by a blank screen (200 ms) was displayed. As dependent variables, we examined the frequency of saccade errors on disorder-specific vs. neutral stimuli, i.e. when the participants failed to look away from the stimuli. First saccade errors correspond to proactive response inhibition, and second saccade errors address reactive response inhibition and corrective behavior [e.g., (35, 36)].

The food and alcohol stimuli have been pretested and have been used in previous studies (18, 25). We used low- to high-calorie food items, e.g. strawberries, fish, pasta, french fries, cakes, and low- to high-alcohol beverages, e.g. beer, wine, cognac, whiskey, and tequila. The neutral stimuli were matched to the disorder-specific stimuli concerning brightness, color and contrast, and depicted household items. To use better visually matched stimuli to the alcohol stimuli, 16 new neutral pictures have been developed and pretested in a pilot study for valence and arousal.

### Procedure

We scheduled two study appointments on two different days, a diagnostic session and the experimental session. In the diagnostic session, we assessed mental disorders using the Structured Clinical Interview for DSM-IV Axis I Disorders (SCID I) (37), while we modified diagnostic criteria to assess AUD and BED according to the novel DSM-5 criteria. The BED group and BED-CG completed the Eating Disorder Examination Questionnaire (EDE-Q) (38), the AUD group and AUD-CG completed the Alcohol Use Disorder Identification Test (AUDIT) (39), and all four groups filled in the Beck Depression Inventory (BDI II) (40) and the Barratt Impulsiveness Scale (BIS-11) (41).

Before the experimental session, the BED group and BED-CG had to fast overnight and received a standardized breakfast to prevent homoeostatic effects. The AUD group and AUD-CG were instructed to be abstinent from alcohol to prevent effects of intoxication, and particularly the participants with AUD had to be free from withdrawal symptoms. At the laboratory, the two experimental tasks were conducted in counterbalanced order, and the participants rated the valence of the presented stimuli on a Likert scale ranging from −5 (extremely unpleasant) to +5 (extremely pleasant).

### Data Analysis

Concerning eye-tracking data, participants with low data quality, i.e. two standard deviations (SD) above mean excluded trials in the free exploration task or above 50% of excluded trials in the antisaccade task were excluded from data analysis. Further, participants with extreme outliers were excluded, because this indicates neglect of the instructions, i.e. two SDs below the average dwell time on both stimulus categories in the free exploration task and two SDs above the average error rate on both stimulus categories in the antisaccade task. Finally, we analyzed 22 BED, 21 BED-CG, 20 AUD, and 18 AUD-CG participants in the free exploration task and 21 BED, 19 BED-CG, 18 AUD, and 18 AUD-CG participants in the antisaccade task.

The statistical analysis was done with SPSS version 24 (42). The sample characteristics, valence rating, and questionnaire data were analyzed with analyses of variance (ANOVAs) or nonparametric tests. The eye-tracking data were analyzed with repeated-measure ANOVAs with the disorder-specific vs. neutral stimuli as the within-subject factor and the four groups as the between-subject factor. As the second saccade errors were not normally distributed, we logarithmized the original values to achieve normal distribution. Due to the pilot character of the study, we computed *post hoc* multiple comparisons according to Bonferroni. Further, we computed bias scores of the eye-tracking variables, i.e. disorder-specific stimuli minus neutral stimuli to compare the groups with *post hoc* tests in univariate ANOVAs.

## Results

### Sample Characteristics

The sample characteristics concerning all four groups are presented in **Table 1**. In the BED group and BED-CG were 23 female participants each, whereas in the AUD group and AUD-CG were 13 female and 8 male participants. Thus, the groups differed in sex ratio with χ^2^(3) = 21.4, *p* < .001. Six participants with BED and six with AUD used antidepressants currently in contrast to none of the controls with χ^2^(3) = 14.0, *p* = .003.

The participants with AUD were abstinent from alcohol since *M* = 3.4 weeks (*SD* = 3.7). Sixteen participants with AUD were currently in an inpatient treatment, four in an outpatient treatment, and one in no treatment. In comparison with AUD-CG, they showed markedly increased AUDIT scores with *F*(1,40) = 255.1, *p* < .001, *e* *^2^* = .86 (AUD *M* = 24.5, *SD* = 6.0, AUD-CG *M* = 3.0, *SD* = 1.2).

The participants with BED had on average *M* = 12.3 (*SD* = 10.0) binge eating episodes in the past 4 weeks according to EDE-Q. Two participants with BED were currently in inpatient treatment, seven were in outpatient treatment, eight participated in a guided self-help program, and six had no treatment. In comparison with the BED-CG, they had significantly increased EDE-Q scores with *U* = 2.0, *p* < .001, *r* = .82 (BED *M* = 3.0, *SD* = 0.8, BED-CG *M* = 0.3, *SD* = 0.4).

### Free Exploration Task

**Figure 1:**
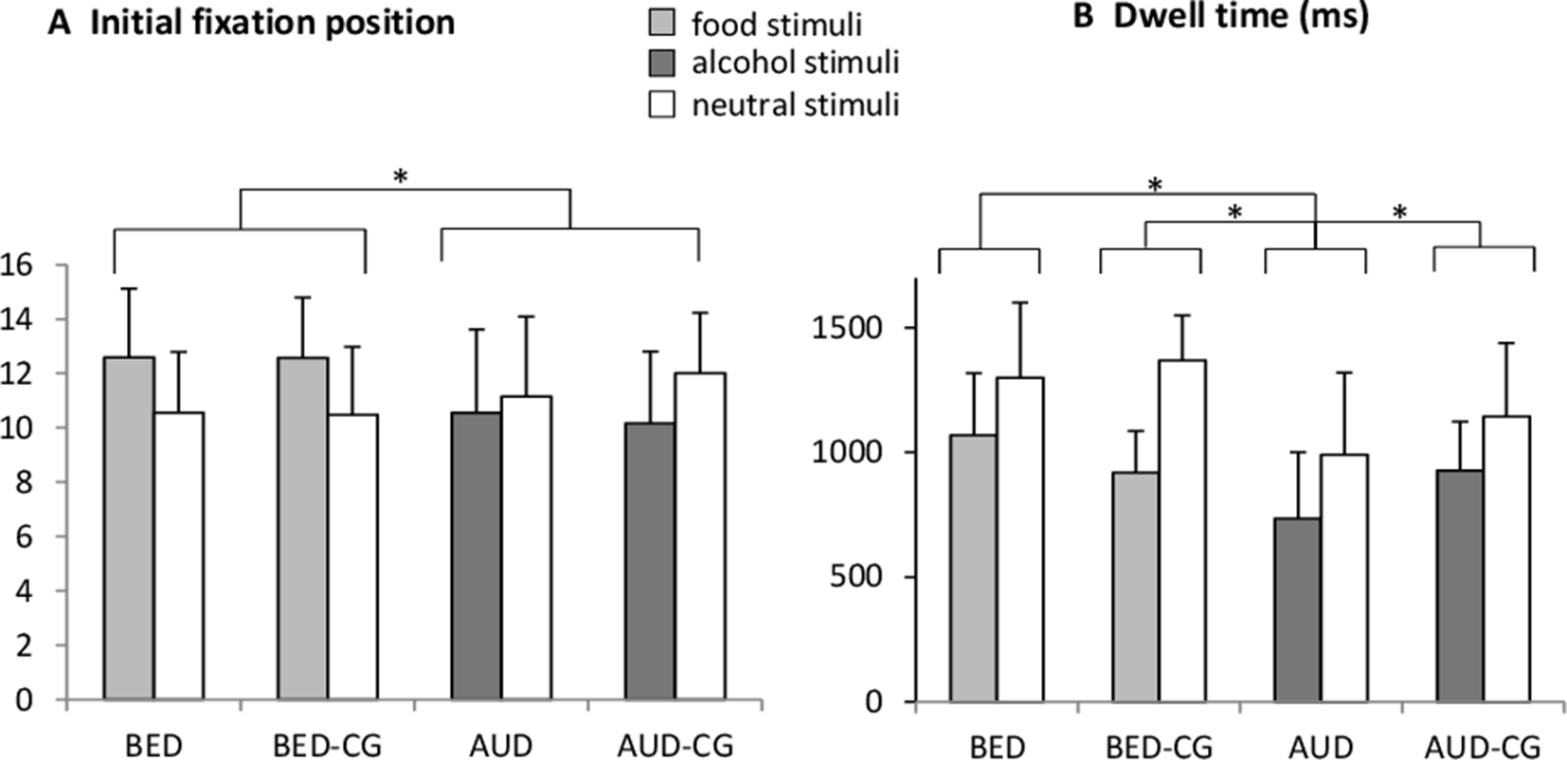
Means and SDs from **(A)** the frequency of the initial fixation position and **(B)** the dwell time (ms) in the free exploration task for food vs. neutral stimuli in the BED group (*N* = 22) and BED-CG (*N* = 21) and alcohol vs. neutral stimuli in the AUD group (*N* = 20) and AUD-CG (*N* = 18). Significant *post hoc* group comparisons (*p* < .05) are marked with an asterisk.

The results of the free exploration paradigm are summarized in **Figure 1**. Concerning the initial fixation position, a significant group × stimulus interaction with *F*(3,77) = 3.3, *p* = .025, *e* ^2^ = .114 emerged. The univariate ANOVA with the bias score shows however *post hoc* no differences between the single groups (*p* > .05). There was also a significant group effect with *F*(3,77) = 3.4, *p* = .030, *e* ^2^ = .109 where *post hoc* tests yielded no differences between the single groups (*p* > .05). We exploratively tested those participants processing food stimuli, i.e. BED and BED-CG together against those processing alcohol stimuli, i.e. AUD and AUD-CG and a significant group × stimulus interaction with *F*(1,79) = 9.4, *p* = .003, *e* ^2^ = .107 as well as a significant group effect with *F*(1,79) = 8.9, *p* = .004, *e* ^2^ = .101 emerged. The interaction indicates that the BED group and BED-CG initially fixated more often on the food stimuli in comparison with neutral stimuli, but the AUD group and AUD-CG initially fixated more often on neutral stimuli in comparison with alcohol stimuli. The group effect is indicating that the AUD group and AUD-CG did less initial fixations on both stimuli categories than the BED group and BED-CG.

Concerning the dwell time, there was a significant stimulus effect with *F*(1,77) = 49.1, *p* < .001, *e* ^2^ = .39 (*M* = 917, *SD* = 250 in disorder-specific stimuli, *M* = 1,207, *SD* = 313 in neutral stimuli), which means that all groups spent overall more time looking at the neutral stimuli. There was also a significant group effect with *F*(3,77) = 13.9, *p* < .001, *e* ^2^ = .35, where *post hoc* tests yielded that the AUD group differed from the AUD-CG (*p* = .019), BED (*p* < .001), and BED-CG (*p* < .001), whereas the BED group did not differ from BED-CG and no other group differences emerged (*p* > .05). This means that the AUD group spent the shortest time looking at the stimuli irrespective of the stimulus category.

### Modified Antisaccade Task

**Figure 2:**
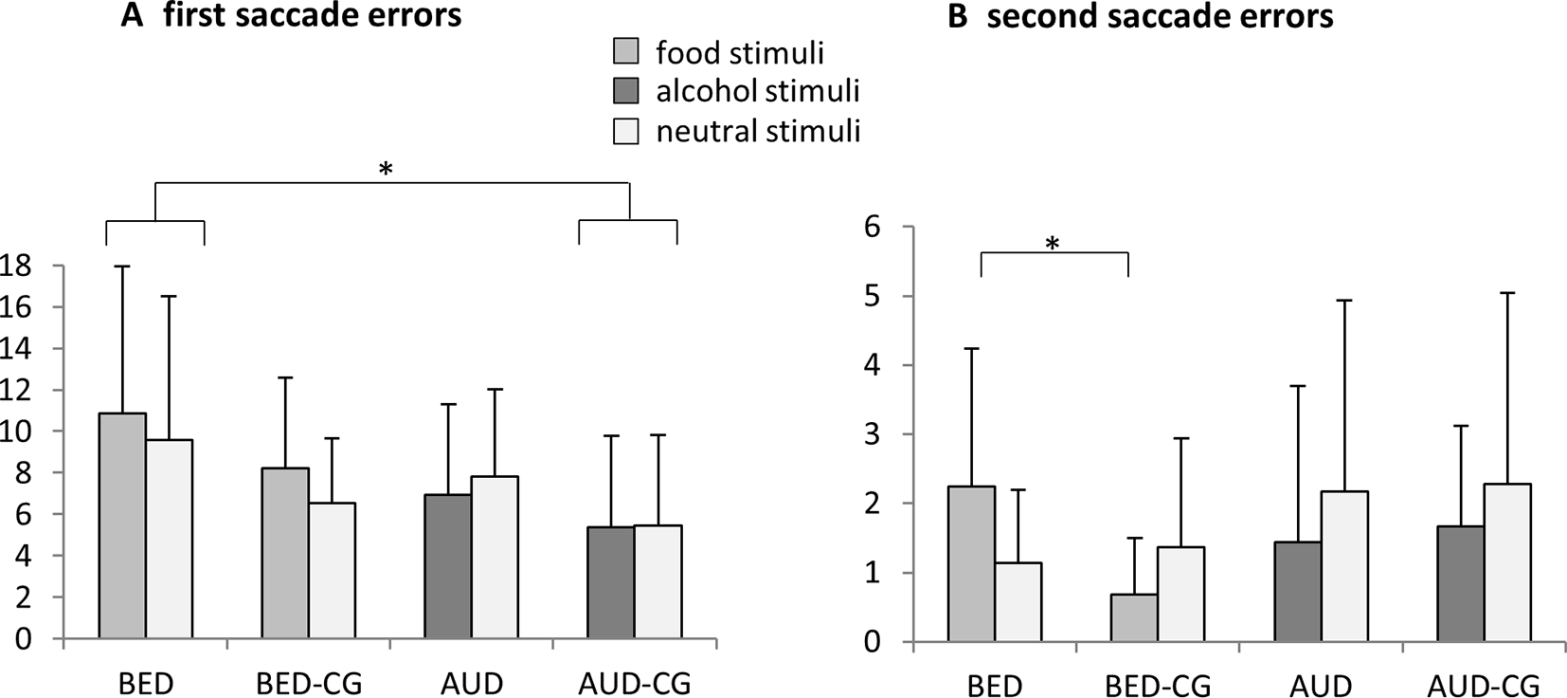
Means and SDs from **(A)** the frequency of first saccade errors and **(B)** the frequency of second saccade errors in the modified antisaccade task for food vs. neutral stimuli in the BED group (*N* = 21) and BED-CG (*N* = 19) and alcohol vs. neutral stimuli in the AUD group (*N* = 18) and AUD-CG (*N* = 18). Significant *post hoc* group comparisons (*p* < .05) are marked with an asterisk.

The results of the modified antisaccade task are summarized in **Figure 2**. Regarding first saccade errors, there was a significant group effect with *F*(3,72) = 3.3, *p* = .024, *e* ^2^ = .123. *Post hoc* tests yielded that the BED group did not differ from BED-CG and the AUD group did not differ from AUD-CG, but the BED group differed significantly from the AUD-CG (*p* = .016). No other group effects emerged (*p* > .05). Accordingly, the BED group made irrespectively of the stimulus category most errors in first saccades, whereas the AUD group did not differ from controls.

Regarding second saccade errors, a significant stimulus × group interaction emerged with *F*(3,72) = 3.4, *p* = .023, *e* ^2^ = .123. The univariate ANOVA with the bias score shows *post hoc* that the BED group differed from the BED-CG (*p* = .040), whereas the AUD group did not differ from the AUD-CG and no other group differences emerged (*p* > .05). Thus, the BED group made more second saccade errors especially for the food stimuli, whereas AUD did not differ from controls.

### Valence Rating of Presented Stimuli

**Figure 3:**
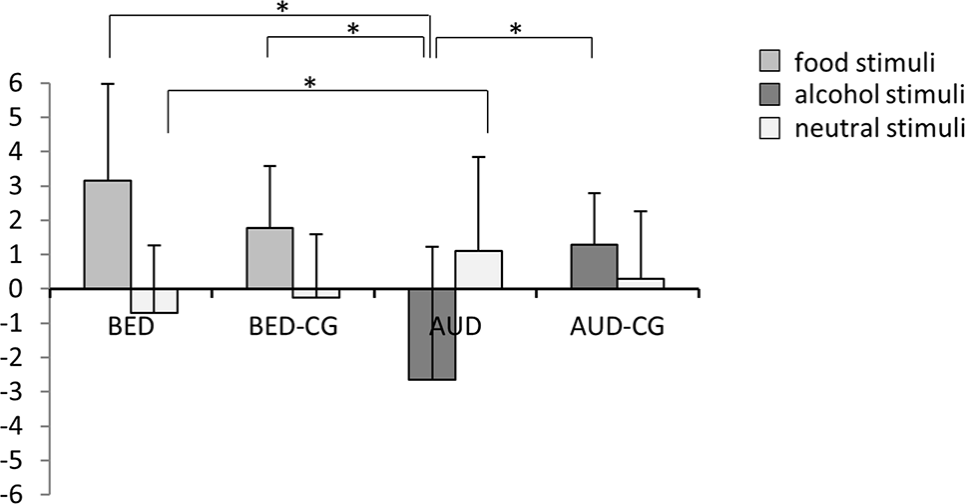
Means and SDs from the valence rating of the stimuli, i.e. food and neutral stimuli in the BED group (*N* = 23) and BED-CG (*N* = 23) and alcohol and neutral stimuli in the AUD group (*N* = 21) and AUD-CG (*N* = 21). Significant *post hoc* group comparisons (*p* < .05) are marked with an asterisk.

The results of the valence rating are summarized in **Figure 3**. The groups differed significantly concerning the rating of disorder-specific stimuli, i.e. food in BED and BED-CG, and alcohol in AUD and AUD-CG with *F*(3,84) = 19.0, *p* < .001, *e* ^2^ = .404. *Post hoc* tests yielded that the BED group and BED-CG did not differ, but the AUD group rated the disorder-specific stimuli as more negative than AUD-CG (*p* < .001), BED (*p* < .001), and BED-CG (*p* < .001). The groups also differed in the rating of neutral stimuli with *F*(3,84) = 2.9, *p* = .040, *e* ^2^ = .094. According to *post hoc* tests, the AUD group did not differ from AUD-CG and the BED group did not differ from BED-CG (*p* > .05), but the AUD group rated the neutral stimuli more positive than the BED group (*p* = .038).

## Discussion

In the present pilot study, we compared participants with BED, participants with AUD and two sex- and age-matched healthy control groups concerning impulsivity towards disorder-specific stimuli. We used food pictures in comparison with neutral pictures in the BED group, and alcohol pictures in comparison with neutral pictures in the AUD group. Our preliminary results suggest that participants with BED and AUD, as compared to healthy controls and neutral stimuli, process disorder-specific stimuli differently. Participants with BED showed decreased inhibitory control in the modified antisaccade paradigm, particularly for disorder-specific (food) stimuli in later processing stages. The BED group did not show increased reward sensitivity for food stimuli in comparison with healthy controls in the free exploration task. Contrary, the participants with AUD seem to generally avoid stimuli in comparison with the other groups. They showed a reduced dwell time on both stimulus categories in the free exploration task, while we had expected increased dwell time on alcohol stimuli, and they did not show inhibitory deficits in the antisaccade task. Also, the AUD group rated the alcohol stimuli as more unpleasant than the other groups and the neutral stimuli as more pleasant than the BED group.

Taking a closer look at the free exploration task, the BED group as well as their respective control group directed initial fixations more often at food stimuli in comparison with neutral stimuli and the AUD and AUD-CG initially fixated more often at neutral stimuli in comparison with alcohol stimuli. Thus, it seems that irrespective of the individual pathology, people in general approach food stimuli, whereas they generally avoid alcohol stimuli at the first sight. Concerning dwell time, all participants spent more time looking at the neutral stimuli in comparison with the disorder-specific stimuli. This might represent a novelty effect or a complexity effect of the neutral stimuli in such a way that more time is needed to identify the neutral stimuli. Further, participants with AUD spent overall less time looking at the stimuli in comparison with the three other groups, particularly in comparison with the AUD-CG. This might be interpreted as a general pattern of avoidance, irrespectively of stimulus content. It might be that this pattern of the AUD group represents a treatment effect, as patients with AUD learn to avoid alcohol stimuli in treatment in the sense of getting more stimulus control. Thus, they could have decided to avoid the presented stimuli *per se*, as they did not know at which position the alcohol stimuli were displayed. The BED group however did not differ from the BED-CG concerning dwell time. In our previous analysis of these groups (25), we found increased gaze duration on food stimuli in comparison with obese and normal weight controls without BED. The different results might be due to a different data analysis strategy, as we also considered the dwell time on the monitor background in this analysis, which we did not consider before. In the valence rating however, participants with BED rated the food stimuli as more pleasant than participants with AUD and the participants with AUD rated the alcohol stimuli as more unpleasant than all other groups. This supports the hypothesis that patients with AUD avoid alcohol stimuli, and that patients with BED show increased reward sensitivity towards food stimuli.

In the modified antisaccade task, the BED group made more errors in first saccades in comparison with the AUD-CG, but not in comparison with the BED-CG and irrespective of the stimulus category. Moreover, they made more errors in second saccades compared with BED-CG, especially when food stimuli have been presented. This pattern speaks for a deficit in inhibitory control in patients with BED that particularly manifests when the patients are confronted with food stimuli. In the previous study from Schag et al. (25), the results for the percentage of first saccade errors were clearer, which might be due to the slightly different outcome that has been used. The participants with AUD, however, did not differ from the AUD-CG in first and second saccade errors, neither when alcohol nor when neutral stimuli were presented. These results show that the participants with AUD are able to inhibit reactions towards alcohol stimuli and are in line with the results from the free exploration task and the valence rating, that patients with AUD seem to dislike alcohol stimuli and try to avoid them.

Taken together, our results are in line with preceding studies that show increased inhibition deficits in BED (13, 20), but they do not match to studies that have shown increased reward sensitivity in BED (19, 20, 33). Moreover, our results concerning AUD do not correspond to our hypotheses based on preceding evidence linking substance abuse with increased reward sensitivity as well as decreased inhibitory control (12, 15, 43). In the direct comparisons of the two patient groups (22–24), impulsivity was increased in patients with addiction and in BED. As Voon and colleagues did not use disorder-specific stimuli, the reason of these different results might be that patients with addictive disorders show more general and less disorder-specific deficits, on the contrary to patients with BED. Further, the different results might be due to different concepts of impulsivity that have been explored, as Voon and colleagues measured impulsivity with tasks addressing delay discounting, decision making and motor response inhibition. Thus, patients with addictions might have more problems with delayed gratification or motor handed rather than oculomotor impulsiveness. This fits with the assumption from Morris and Voon (14) that patients with addictive disorders and BED show increased impulsivity, but in different facets of impulsivity.

Due to the pilot character and transdiagnostic approach of this study comparing two patient groups directly, several limitations of this study must be kept in mind. First, the study might have been somewhat underpowered to detect group effects as the results concerning initial fixation position indicate, though we overall detected differences between groups. Another limitation is that the participants with AUD had to be abstinent before the eye-tracking tasks to avoid effects of intoxication, whereas the participants with BED still showed binge eating and got a standardized breakfast to control individual hunger levels. This might have impacted the results though other studies comparing these two patient groups used the same design (22–24). Though we controlled BMI and matched for gender and age in the patient groups and respective control groups, different sex ratios with only females in the BED and BED-CG, and with males and females in the AUD and AUD-CG might have biased the results, because males are generally more impulsive (44). However, in this study the BED group with only females scored highest on the impulsivity questionnaire (BIS-11). Another point is that the intake of antidepressants might have affected the results, though a systematic review concludes that they have no adverse impact on oculomotor responses (45). Further, we only presented the disorder-specific and corresponding neutral stimuli to each patient group and its respective control group, and did not present all stimuli to all groups. This also might have biased the results, though we pretested the stimulus material thoroughly.

The strengths of this pilot study include the direct comparison of participants with BED and AUD using established eye-tracking paradigms, and the use of two age- and sex-matched control groups for each patient group. Moreover, this is to the best of our knowledge the first eye-tracking study to investigate impulsive processing of disorder-specific stimuli in participants with AUD and BED. Further strengths include the use of standardized diagnostic interviews and controlling for hunger levels and alcohol intake prior to the experiment.

To conclude, these preliminary results should be interpreted with caution, but indicate that patients with AUD and BED show different deficits in impulsivity: Patients with BED might fail to inhibit reactions, whereas patients with AUD might avoid stimuli in a more stimulus-independent way. Thus, the term “food addiction” might be misleading as the addiction concept is not one-to-one transferable to BED. In practical terms, whereas patients with BED have to learn how to eat regularly in average-sized portions, patients with AUD have to learn how to avoid alcohol drinking to be totally abstinent.

## Data Availability Statement

Datasets are available on request from the corresponding author.

## Ethics Statement

This study was carried out in accordance with the recommendations of the ethics committee of the Medical Faculty at the Eberhard Karls University Tübingen, Germany with written informed consent from all subjects. All subjects gave written informed consent in accordance with the Declaration of Helsinki. The protocol was approved by the ethics committee of the Medical Faculty at the Eberhard Karls University Tübingen, Germany.

## Author Contributions

KS, MR-S, AB, and KG designed the study and wrote the protocol. MR-S performed the experiments. MR-S and KS analyzed the data. AB and SZ contributed the study materials. FW contributed to the patient recruitment. KS and KG drafted the manuscript. MR-S, FW, AB, and SZ critically revised the manuscript. All authors have approved the final manuscript.

## Funding

Funding for this publication was provided by DFG Open Access Publishing Fonds.

## Conflict of Interest

The authors declare that the research was conducted in the absence of any commercial or financial relationships that could be construed as a potential conflict of interest.
